# Desmosomal Component Expression in Normal, Dysplastic, and Oral Squamous Cell Carcinoma

**DOI:** 10.1155/2010/649731

**Published:** 2010-03-18

**Authors:** Nagamani Narayana, Julie Gist, Tyler Smith, Daniel Tylka, Gavin Trogdon, James K. Wahl

**Affiliations:** Department of Oral Biology, University of Nebraska Medical Center College of Dentistry, 40th and Holdrege, Lincoln, NE 68583, USA

## Abstract

Squamous cell carcinoma (oral SCC) is the most common oral cancer in the U.S., affecting nearly 30,000 Americans each year. Despite recent advances in detection and treatment, there has been little improvement in the five-year survival rate for this devastating disease. Oral cancer may be preceded by premalignant disease that appears histologically as dysplasia. Identification of molecular markers for cellular change would assist in determining the risk of dysplasia progressing to oral squamous cell carcinoma. The goal of this study was to determine if any correlation exists between histological diagnosed dysplasia and OSCC lesions and altered expression of desmosomal cell-cell adhesion molecules in the oral epithelium. Our data showed that oral SCC tissue samples showed decreased immunoreactivity of both desmoplakin and plakophilin-1 proteins compared to normal oral epithelium. Furthermore, significant decrease in desmoplakin immunoreactivity was observed in dysplastic tissue compared to normal oral epithelium. In contrast, the level of desmoglein-1 staining was unchanged between samples however desmoglein-1 was found localized to cell borders in oral SCC samples. These data suggest that changes in expression of desmoplakin and plakophilin-1 may prove to be a useful marker for changes in tissue morphology and provide a tool for identifying pre-neoplastic lesions of the oral cavity.

## 1. Introduction

Oral cancer affects 3% of the United States population and it is estimated that 35,000 new cases will be diagnosed this year [1]. Despite recent advancements in detection and treatment of oral SCC, survival has only modestly improved in the past 30 years (reviewed in [2]). Changes in tumor cell migration and interactions with the extracellular environment have been demonstrated to promote the progression of many solid tumors. Alterations in adhesive characteristics of cancer cells allow rapidly growing tumor cells to detach from their neighbors, infiltrate the underlying stroma, and disseminate to distant sites in the body establishing a tumor metastasis. Therefore understanding the changes in adhesion molecule expression is important for determining the invasive capacity of cells in a tissue and predicting the likelihood of metastasis. It has been proposed that areas of oral dysplasia may progress to oral SCC over time [3, 4] and therefore can be considered premalignant lesions. However, diagnosing dysplasia using tissue morphology is subjective and depends upon the training and experience of the oral pathologist. Therefore, identification of molecular markers for cellular change would assist in recognition of premalignant lesions and assist in determining the risk of dysplasia progressing to malignancy. Characterization of novel markers would also assist in earlier diagnosis and thereby improve the prognosis of oral cancer.

Desmosomes are the most prominent cell-cell junctional complex in stratified squamous epithelial tissues. Loss of desmosomes in various types of carcinomas is associated with increased migratory capacity of the tumor cells [5–7]. The transmembrane core of the desmosome is comprised of single pass desmosomal cadherins (desmogleins and desmocollins) that are believed to interact heterotypically and homotypically in the extracellular space to mediate cell-cell adhesion [8, 9]. In addition to the desmosomal cadherins, the recently identified tetraspan protein, PERP has also been shown to localize to the desmosome and affect desmosome assembly in keratinocytes [10]. The cytoplasmic domain of the desmosomal cadherins associates directly with several desmosomal plaque proteins, including plakoglobin and plakophilins that in turn recruit the keratin intermediate filament cytoskeleton via interactions with desmoplakin [11]. Assembly of the desmosomal junction allows the keratin intermediate filament cytoskeleton to stretch across cells and provide epithelial tissues a mechanism to withstand mechanical stress. Inactivation of specific desmosomal adhesion complexes by autoimmune sera as seen in pemphigus vulgaris or pemphigus foliaceus results in epidermal blisters [12]. 

Inherited mutations in desmosomal genes have been identified that result in various skin, hair, and heart defects (reviewed in [13, 14]). Mutations in plakophilin-1 are associated with ectodermal dysplasia and skin fragility syndrome [15] while mutations in desmoglein-1 are associated with striate palmoplantar keratoderma. Mutations in desmoplakin and plakophilin-2, two genes encoding desmosomal components expressed in the heart, have been implicated in the development of arrhythmogenic right ventricular cardiomyopathy (ARVC) [16]. ARVC patients exhibit fibro-fatty replacement of the heart muscle which can result in sudden cardiac death. Often patients harboring desmoplakin mutations can also exhibit defects in hair and skin due to disruption of desmosomes in these epithelial tissues.

Given these findings, we hypothesize that altered expression and/or localization of the desmosomal proteins may result as cells become dysplastic and eventually progress to squamous cell carcinoma. While many studies have described the localization and expression of desmosomal components in skin and skin tumors, relatively little is known regarding desmosomal component expression in tumors arising from the oral mucosa. In this study we examined the expression of two desmosomal plaque proteins, desmoplakin and plakophilin-1. Desmoplakin is found in desmosomes in all the living layers of the epidermis while plakophilin-1 is most highly expressed in differentiated layers. Additionally, we examined the expression of the differentiation specific desmosomal cadherin, desmoglein-1. In this study we hypothesized that changes in differentiation specific components are more likely to exhibit changes in expression between normal and dysplastic samples. These changes are likely to be maintained in oral SCC samples. 

## 2. Materials and Methods

### 2.1. Tissue Procurement and Immunostaining

Archival tissue sections from The UNMC Oral Pathology service were obtained with approval from the UNMC Institutional Review Board. Eight of normal (oral buccal mucosa) fifteen histologically confirmed dysplasia samples and fifteen oral squamous cell carcinoma samples were used for analysis. All paraffin embedded tissue sections cut into 5 *μ*m sections and collected onto charged Superfrost slides (Electron Microscopy Sciences, Hatfield, PA.). Formalin fixed paraffin embedded sections were dewaxed using xylene and rehydrated through a graded alcohol series and water. Antigen retrieval was achieved by microwave treatment for 5 minutes in freshly prepared 10 mM Sodium citrate (pH 6.0). Tissues were incubated in blocking buffer (1 x phosphate buffered saline, 0.1% Triton x-100, and 1% bovine serum albumin) for 30 minutes prior to incubation with primary antibodies overnight at 4°C. Excess primary antibodies were removed by extensive washing with 1 x phosphate-buffered saline. Tissues were incubated with appropriate FITC-conjugated antimouse secondary antibodies and mounted in vectashield mounting media containing DAPI (Vector Laboratories, Burlingame, CA). Images were collected on a Zeiss axiovert 200 M microscope and axiocam CCD camera using SlideBook software from Intelligent Imaging Innovations (Denver, CO.)

### 2.2. Antibodies

Mouse monoclonal antidesmoplakin (10F6), plakophilin-1, and desmoglein-1 were generated in our laboratory as previously described [17, 18]. Antidesmoplakin antibody 10F6 recognizes the carboxy terminal domain of human desmoplakin (AA 1960-2151). Generation of antiplakophilin-1 and antidesmoglein-1 monoclonal antibodies has been described previously [18, 19].

### 2.3. Evaluation of Staining Behavior

Immunostaining was evaluated in a semiquantitative system based on the scoring of at least three independent evaluators. Scoring was based on overall staining throughout the tissue rather that staining at one field of view. As a negative control, normal oral mucosa was processed without the primary antibodies and this signal intensity was determined to be background. A numerical score for each sample was assigned based on the following scale. Intense cell border signal and relatively weak cytoplasmic signal was given a score of “3”, moderate cell border and cytoplasmic staining intensity was given a score of “2”, overall weak staining intensity was scored a “1”, and staining intensity similar to background levels was scored “0”. For plakophilin-1 immunostaining, the observers were instructed to judge the nuclear signal together with the cytoplasmic signal to arrive at an overall signal intensity score since the nuclear signal was often heterogeneous throughout a given tissue sample. Statistical analysis of scores was carried out by ANOVA analysis and significant differences were determined by Kruskal-wallis multiple comparisons.

## 3. Results and Discussion

**Figure 1:**
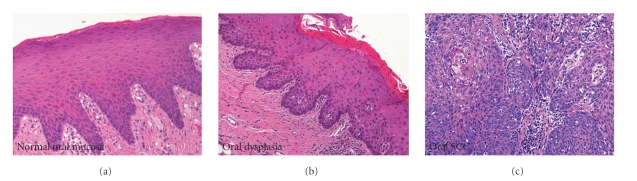
Morphologic evaluation of normal oral mucosa (a), dysplastic oral mucosa (b), and oral squamous cell carcinoma (c). Representative sections were stained with Hematoxylin and eosin to verify the initial diagnosis of the tissue blocks used in the present study.

To begin our analysis we selected a panel of previously diagnosed dysplasia and oral SCC tissue samples available as part of the UNMC oral pathology biopsy service within the college of dentistry. We selected fifteen dysplasia samples and fifteen oral SCC samples to be compared to eight normal oral mucosa samples. Dysplastic tissues were chosen based on the presence of basal cell layer hyperplasia, cellular pleomorphism, increased mitotic figures, and disorganization of stratification within the epithelium compared to normal oral mucosa (Figure 1(b)). For the purposes of this study we chose not to further stratify the dysplasia samples due to the high degree of subjectivity in the diagnosis of these samples. Oral SCC samples corresponded to moderately differentiated squamous cell carcinomas as diagnosed by oral pathologists within the UNMC biopsy service (Figure 1(c)). Normal oral mucosa was obtained from samples exhibiting underlying fibroma with normal appearing surface epithelium (Figure 1(a)). Hematoxylin and eosin staining of the tissue was used to reconfirm the diagnosis and representative sections are shown in Figure 1. 

Previous reports have demonstrated that desmosomal component expression is often reduced or absent in oral SCC when compared to normal epithelium [5, 20]. For our analysis we chose to include dysplastic samples to determine if loss of desmosomal adhesion is an early event in the progression to squamous cell carcinoma. We stained a panel of tissues using monoclonal antibodies specific for human desmosomal components (desmoplakin, plakophilin-1, and desmoglein-1) and compared the staining patterns of dysplastic and oral SCC samples to that of normal oral mucosa. 

**Figure 2:**
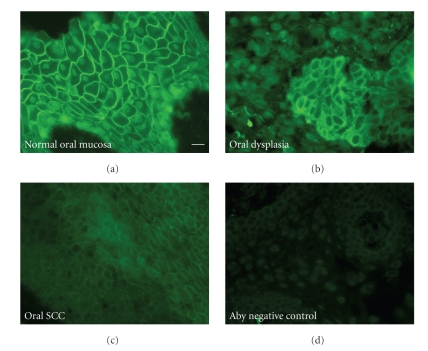
Representative antidesmoplakin staining of oral tissues. Antidesmoplakin monoclonal antibody (10F6) was used to stain normal oral mucosa (a), dysplastic oral mucosa (b), and oral squamous cell carcinoma (c). Normal oral mucosa processed in the absence of primary antibody serves as a negative control (d). The scale bar in panel A corresponds to 20 *μ*m.

Desmoplakin is an obligatory component of the desmosomal plaque that has been shown to play an essential role in recruiting the keratin intermediate filament cytoskeleton to sites of cell-cell adhesion. As expected, desmoplakin staining in normal oral mucosa displayed an intense staining pattern present at cell-cell borders in all the differentiated layers of epidermis (Figure 2(a)). Some diffuse cytoplasmic signal was observed; however this signal was minor compared to the cell border staining. Some faint nuclear signal was also present that was determined to be background signal arising from the secondary antibody since this signal was also seen in the negative control samples in which the antibody was omitted (Figure 2(d)). 

Desmoplakin immunostaining of oral dysplastic tissues revealed a disruption in the desmosomal localization of desmoplakin resulting in diffuse cytoplasmic localization. In addition, there was a decrease in the overall intensity of the antidesmoplakin signal (Figure 2(b)). Areas of relatively normal desmoplakin localization could be seen but these regions were small and did not extend throughout the dysplastic tissues (data not shown). Staining of oral SCC samples with antidesmoplakin antibodies revealed relatively low protein expression in several samples; however the small amount of desmoplakin that was present could be seen localized at cell borders in a pattern similar to that seen in normal tissues (Figure 2(c)). Most oral SCC samples displayed no antidesmoplakin immunoreactivity and were scored as negative. 

Semiquantitative scoring was performed to assess the desmoplakin signal intensity across the panel of tissues. At least three independent observers were trained to recognize normal cell border-associated desmoplakin staining in normal tissues and negative background signal associated with a negative control tissue. Identifiers were removed from the slides and scores were recorded. Desmoplakin staining in normal oral mucosa samples scored highest while staining in dysplastic samples and oral SCC samples scored significantly lower (Figure 5). These data suggest that loss of antidesmoplakin immunoreactiviy is detectable during the transition from normal to dysplasia in the oral cavity. 

**Figure 3:**
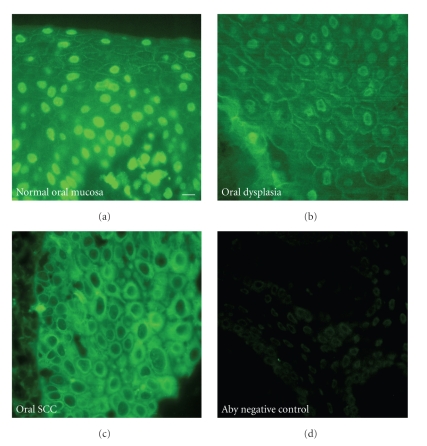
Representative antiplakophilin-1 staining of oral tissues. Antiplakophilin-1 monoclonal antibody (14B11) was used to stain normal oral mucosa (a), dysplastic oral mucosa (b), and oral squamous cell carcinoma (c). Normal oral mucosa processed in the absence of primary antibody serves as a negative control (d). The scale bar in panel A corresponds to 20 *μ*m.

Plakophilins are a family of armadillo repeat proteins that play an important role in assembly and maintenance of the desmosome [14, 21]. Interestingly, plakophilin-1 has also been identified as a nuclear protein in cultured cells derived from stratified epithelial tissues [22, 23]. Plakophilin-1 has been reported to be highly expressed in the most differentiated layers of stratified squamous epithelium such as skin [7, 24]. Unlike plakophilin-1 localization in the keratinizing epithelium of the skin, plakophilin-1 localized to all the living layers of the epithelium in the oral mucosa (Figure 3(a)). This difference in expression is likely to reflect a difference between keratinized and nonkeratinized tissues. 

In addition to the expected cell border localization of plakophilin-1 in normal oral mucosa, we also observed intense nuclear localization of plakophilin-1. The cell border signal seen for plakophilin-1 resembled that of desmoplakin although the intensity was somewhat reduced. The majority of plakophilin-1 signal seen in normal samples was concentrated in the nucleus (Figure 3(a)) and often obscured the signal seen at the cell periphery. Nuclear localization of plakophilin-1 in cultured cells has been previously reported [22, 25, 26]. Nuclear localization of plakophilin-1 in formalin fixed tissues is not homogenous throughout the individual tissues. Some areas of the tissue were observed in which no nuclear plakophilin-1 was observed while strong cell border staining was present. Currently, the nuclear function of plakophilin-1 is unknown. Plakophilin-1 immunostaining of dysplasia samples often revealed a decrease in overall intensity, especially in the nuclear plakophilin-1 pool (Figure 3(b)). Nuclear plakophilin-1 was only rarely and weakly observed in dysplastic and oral SCC samples. Plakophilin-1 localization in oral SCC samples was most often diffusely localized in the cytoplasm with little to no nuclear signal (Figure 3(c)). Complete loss of cell border association of plakophilin-1 was often seen in the oral SCC samples (data not shown).

Scoring of the antiplakophilin-1 signal in these tissues revealed an overall slight decrease in the dysplastic tissues compared to normal oral mucosa although this change was not significant. Comparison of plakophilin-1 staining in oral SCC samples to staining in normal oral mucosa revealed a significant decrease in plakophilin-1 immunoreactivity between these tissue samples (Figure 5). Consistent with previous reports, plakophilin-1 staining is decreased in SCC compared to normal tissue [7, 24, 27]; however plakophilin-1 is not significantly decreased in dysplastic tissues.

**Figure 4:**
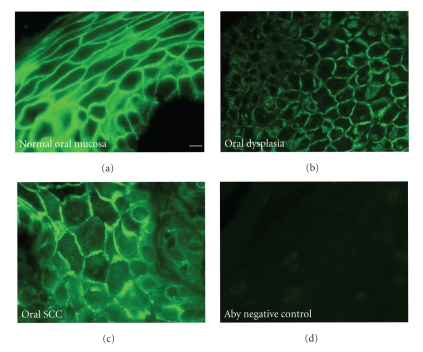
Representative anti-desmoglein-1 staining of oral tissues. Anti-desmoglein-1 monoclonal antibody (27B2) was used to stain normal oral mucosa (a), dysplastic oral mucosa (b), and oral squamous cell carcinoma (c). Normal oral mucosa processed in the absence of primary antibody serves as a negative control (d). The scale bar in panel A corresponds to 10 *μ*m.

**Figure 5:**
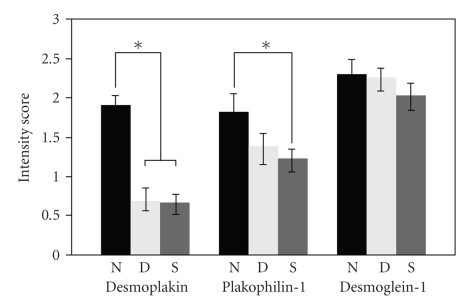
Semiquantitative scoring of desmosomal component expression. Relative intensity of desmoplakin, plakophilin-1, and desmoglein-1 was scored by three observers and the average score is presented (+ or − the standard error) for normal oral mucosa (N), dysplastic epithelium (D), and oral squamous cell carcinoma (S) (* :  *z* value >2.394 indicate significant difference).

Desmoglein-1 is a transmembrane desmosomal cadherin most highly expressed in the differentiated layers of the epidermis. In our oral mucosa samples, this differentiation specific desmosomal cadherin was highly expressed in all the living layers of normal oral mucosa unlike skin where desmoglein 1 expression is restricted to the most differentiated cell layers [28]. Interestingly, we observed robust desmoglein-1 signal in dysplastic tissues and in oral SCC samples. The staining intensity of the antidesmoglein-1 signal was often slightly reduced; however we consistently observed significant desmoglein-1 protein in all of the samples we observed (Figure 4). Although desmoglein-1 localization in oral SCC was at cell borders, it was possible to also observe some cytoplasmic punctuate signal near cell borders. This altered localization was consistently observed in all the oral SCC samples. Scoring of the antidesmoglein-1 staining in these tissues did not reveal a significant change in anti-desmoglein-1 immunoreactivity between our samples.

Although our sample size is relatively small, we are able to detect clearly significant changes in desmoplakin immunoreactivity between normal and dysplastic oral epithelium suggesting that disruption of desmosomal adhesion may be an early event in the progression to oral SCC. In addition, changes in plakophilin-1 expression appear at a later stage compared to changes seen for desmoplakin immunoreactivity, possibly in response to altered keratin intermediate filament attachment at sites of cell-cell contact. 

In our samples, desmoglein-1 immunoreactivity was not significantly altered between normal and oral SCC samples. This finding is in disagreement with a recent study that showed an inverse correlation of desmoglein-1 expression and poor prognosis of head and neck squamous cell carcinoma patients [29]. The relatively small size of our sample pool may explain the differences observed between the two studies. The distribution of desmoglein-1 was often disrupted and was not concentrated at cell borders but was rather diffuse throughout the cells, most likely on the cell surface (Figure 4(c)). Based on the loss of the desmosomal plaque proteins, desmoplakin and plakophilin-1, diffuse localization of desmoglein-1 is not unexpected. Examination of these cell adhesion markers, particularly desmoplakin, in dysplastic tissues may provide a good marker of tissues at increased risk for progression to oral SCC.
